# Systemically Infused Mesenchymal Stem Cells Show Different Homing Profiles in Healthy and Tumor Mouse Models

**DOI:** 10.1002/sctm.16-0204

**Published:** 2017-02-16

**Authors:** Chengying Xie, Zhangru Yang, Yuanzhen Suo, Qianqian Chen, Dan Wei, Xiaofu Weng, Zhengqin Gu, Xunbin Wei

**Affiliations:** ^1^ Med‐X Research Institute and School of Biomedical EngineeringShanghaiChina; ^2^ Department of UrologyXinhua Hospital, School of Medicine, Shanghai Jiao Tong UniversityShanghai 200092China

**Keywords:** Mesenchymal stem cells, Homing, Hepatocellular carcinoma, Tumor xenograft model, In vivo flow cytometry

## Abstract

Bone marrow‐derived mesenchymal stem cells (MSCs) can localize in injured, inflamed, and cancerous tissues after systemic infusion. However, the dynamic homing profile of MSCs in the peripheral blood is not well characterized. Here, using in vivo flow cytometry to noninvasively monitor the dynamics of fluorescence‐labeled cells, we found different clearance kinetics of systemically infused MSCs between healthy and tumor mouse models. The circulation times of MSCs in healthy mice and mice with subcutaneous tumors, orthotopically transplanted liver tumors, or metastatic lung tumors were 30, 24, 18, and 12 hours, respectively, suggesting that MSCs actively home to tumor environments. MSCs infiltrated into hepatocellular carcinoma (HCC) sites and preferentially engrafted to micrometastatic regions both in vivo and in vitro. The expression of epidermal growth factor, CXCL9, CCL25, and matrix metalloproteinases‐9 by HCC cells differed between primary tumor sites and metastatic regions. By characterizing the homing profiles of systemically perfused MSCs under physiological and cancerous conditions, these findings increase our understanding of the migration of MSCs from the circulation to tumor sites and constitute a basis for developing MSC‐based anti‐cancer therapeutic strategies. Stem Cells Translational Medicine *2017;6:1120–1131*

**Significance Statement**

Bone marrow mesenchymal stem cells (MSCs) are a rare population of nonhematopoietic stromal cells with tropism to damaged or tumor sites. However, the dynamic homing profile of systematically infused MSCs in vivo is not illustrated. Here, we have observed that MSCs possess different circulation times between healthy and tumor mouse models. After depleted from blood stream, MSCs migrate to periphery of tumor regions and preferentially infiltrate into micrometastasis both in vivo and in vitro. The expression of cytokine epidermal growth factor, CXCL9, CCL25, and MMP9 were differently expressed between primary tumor and metastasis sites, especially when cocultured with MSCs, suggesting a crosstalk between MSCs and tumor cells. These results first present the dynamic homing profile of MSCs in periphery blood under physiological and tumor conditions. It helps us understand the homing mechanism of systematically administered MSCs, and design MSCs‐based vehicles to deliver therapeutic agents for targeted tumor therapy.

## Introduction

Hepatocellular carcinoma (HCC) ranks fifth worldwide among all malignancies and is the third leading cause of cancer mortality 1. Tumors are often considered “wounds that never heal” 2, consisting of both cancer cells and diverse cells of origin from different tissue and organs that facilitate a microenvironment conducive to carcinogenesis 3. Tumor microenvironments mainly include angiogenic vascular cells, infiltrating immune cells, and cancer‐associated fibroblastic cells 4. Cancer‐associated fibroblastic cells may arise from fibroblasts residing in local tissues and periadventitial cells including pericytes, vascular smooth muscle cells, endothelial cells, and bone marrow‐derived cells including various stem cells 5.

Mesenchymal stem cells (MSCs) are nonhematopoietic multipotent stem cells that can be isolated from various tissues such as bone marrow, adipose tissue, fetal lung tissue, placental tissue, and umbilical cord blood 6. Recently, several studies suggest that MSCs are recruited by neoplasia as a potential source of cancer‐associated fibroblastic cells to create a favorite niche that promotes carcinogenic progression by paracrine secretion to increase metastatic abilities 7, 8, recruit and activate infiltrating immune cells to provide mitogenic signals to cancer cells 5, stimulate angiogenesis or remodel neovascularization 9, and promote tumor growth 4. Systemically infused MSCs localize within injured, inflamed, and cancerous tissues, suggesting that MSCs could serve as a cellular drug delivery system for multiple applications. MSCs have been genetically engineered to express specific cytokines to treat cancer due to their innate tropism for tumor environments 10, 11, 12, 13. However, the efficiency and speed with which systemically infused MSC traffic to specific sites is unclear. Furthermore, the mechanisms underlying the homing process and recruitment of MSCs into tumors and their potential role in malignant tumor progression are not well known.

There are three main approaches to detecting MSCs after intravenous infusion: (a) ex vivo histological analysis including tissue sectioning, species mismatch 14, real‐time polymerase chain reaction (RT‐PCR) 15, and live tissue imaging 16; (b) imaging methods to visualize cell homing in different organs, including bioluminescence imaging 17, single‐photon emission computed tomography, nuclear scintigraphic tracking 18, positron emission tomography, intravital microscopy 14, and magnetic resonance imaging 19; and (c) quantification of MSCs in peripheral blood by conventional ex vivo flow cytometry 16. Each approach has advantages and disadvantages. For instance, histological analysis requires sacrificing many animals at multiple time points, and sampling is limited when only certain parts of the tissue or organ are harvested and analyzed. Positron emission tomography and nuclear scintigraphic tracking require radioisotope tagging to quantify the relative level of radioactivity in excised tissues and organs within a short period of time. Tracking cells by magnetic resonance imaging requires labeling with contrast agents to visualize the cells, and false positives are observed even if the grafted cells die. Also, using conventional ex vivo flow cytometry to detect MSCs in the circulation requires frequent blood sampling and short testing windows.

However, in vivo flow cytometry can be used to quantify fluorescence‐labeled cells in the circulation without extracting blood samples, allowing noninvasive and continuous assessment of larger blood volumes than ex vivo flow cytometry 20. This technique has been used to detect and quantify rare circulating cells in vivo, such as circulating tumor cells 21, 22, hematopoietic stem cells 23, lymphocytes 24, and apoptotic cells 25.

Here, we monitored MSCs transfected with green fluorescent protein (GFP) by in vivo flow cytometry to study the homing time course and fate of MSCs after systemic delivery into healthy mice and three types of malignant HCC mouse models. We found that the homing profiles of MSCs in the peripheral blood differed between healthy mice and HCC tumor mouse models and that MSCs preferentially homed to micro metastatic regions over primary tumor sites. Moreover, our results suggest that MSCs show different patterns of responses to paracrine factors secreted by HCC cells in primary tumor sites and metastatic regions both in vitro and in vivo.

## Materials and Methods

### Animals

Animal care and experimental protocols were in accordance with guidelines established by the Shanghai Medical Experimental Animal Care Commission. Balb/c nude mice (male, 6 weeks old, 20 ± 2 g) were purchased from Shanghai Laboratory Animal Center. All procedures were approved by the Ethical Committee of Animal Experiments of Med‐X Research Institute and the School of Biomedical Engineering at Shanghai Jiao Tong University.

### Isolation and Expansion of MSCs

Balb/c mice (2–3 weeks old) were decapitated, and the femur and tibia were carefully removed. Bone marrow cells were flushed with a syringe (27‐gauge) inserted into one end of the bone and cultured at a density of 3 × 10^5^ cell/cm^2^ with low‐glucose Dulbecco's modified Eagle's medium (DMEM; Gibco) containing 15% fetal bovine serum (FBS; Gibco) and 1% penicillin/streptomycin (Gibco). After 2 hours, nonadherent cells were removed via medium changes, which were repeated every 3 days until the cultured cells reached 70%–80% confluence. Cells were then digested with TrypLE Express (Gibco) for 2 minutes and passaged. All experiments used MSCs at passage 5–8.

To evaluate their multipotent differentiation potential, MSCs were incubated at passage 5 in a 12‐well plate at a density of 5 × 10^3^ cells/cm^2^ with osteogenesis differentiation medium (Gibco) or at a density of 1 × 10^4^ cells/cm^2^ with an adipogenesis differentiation kit (Gibco) for 3 weeks. According to the manufacturer's instructions, other MSCs were seeded at a higher density of 1 × 10^7^ cells per milliliter to formulate a micromass and cultured with a STEMPRO chondrogenesis differentiation kit (Gibco) with medium changes every 3 days for 2 weeks. Osteoblasts were stained using Alizarin Red S (Sigma), adipocytes were stained with Oil Red O (Sigma), and chondrogenesis pellets were stained with Toluidine Blue (Sigma).

### Conventional Ex Vivo Flow Cytometry

MSCs were trypsinized and resuspended at a density of 1 × 10^5^ cells in 100 μl cold PBS and stained with 1 μg antibody against CD29, CD44, stem cell antigen (Sca‐1), CD45, CD34, or CD31 (eBioscience, San Diego) at 4°C for 40 minutes. After fixation in dark conditions, MSCs were analyzed by a flow cytometer (FACS Aria II, Becton, Dickinson and Company, Franklin Lakes, NJ) to identify cell surface markers and purity. To evaluate the sensitivity and accuracy of the data obtained from in vivo flow cytometry, blood samples were analyzed by BD FACSCalibur and FlowJo 7.0 software (Becton, Dickinson and Company). After MSC infusion through the tail vein, 150 μl blood samples were collected through the orbital venous plexus of mice at different time points.

### Cell Culture and Transfection Procedure

A human HCC cell line, HCCLM3, with high metastatic potential was established at the Liver Cancer Institute, Zhongshan Hospital, Fudan University 26 and maintained in high‐glucose DMEM with 10% FBS. HCCLM3 cells were transfected with linearized pTurboRFP (Evrogen) or pIRES‐EGFP (Clontech) using Lipofectin2000 reagent (Invitrogen). Stable expression of red fluorescent protein (RFP) or GFP was achieved by selecting with 800 μg/ml neomycin. HepG2 and 293T cells were cultured in DMEM with 10% FBS. MSCs were transfected by adenoviruses with pDOV‐mCMV‐MCS‐EGFP (Obio Technology Co., ltd.) at 5,000 viral particles per cell for 60 hours in cell culture medium. The transfection efficiency of MSCs was above 95% based on conventional ex vivo flow cytometry results.

### Cell Migration Assay

The ability of MSCs to migrate to HCCLM3 cells was confirmed by in vitro cell migration assay. HCCLM3, HepG2, and 293T cells were seeded at a density of 2.5 × 10^5^ cells in 800 μl culture medium in the bottom well of a transwell plate (8‐μm pore size polycarbonate membrane, Becton, Dickinson) for 24 hours. Then, 4 × 10^4^ MSCs in 300 μl culture medium were added to the top well. After coculture in an incubator for 24 hours, MSCs remaining on the top side of the membrane were removed by a cotton swab, and migrated cells on the bottom side were fixed. MSCs were stained with 0.5% crystal violet (Sigma‐Aldrich). Twenty fields per well were randomly selected and counted at ×10 magnification with a microscope (Leica). Data were analyzed by Image Pro Plus 6.0 software.

For coculture studies, 1 × 10^5^ MSCs were seeded on the top of a 0.4‐μm pore size 6‐well transwell plate (Corning Costar, Cambridge, MA). GFP‐HCCLM3 cells were seeded at a density of 5 × 10^5^ cells on the bottom to prevent contact between tumor cells and MSCs. After incubation for 24 hours, total RNA was extracted from MSCs or GFP‐HCCLM3 cells using Trizol reagent (Invitrogen).

### Tumor Cell Extraction From Tissue and Culture

All isolation procedures were performed under aseptic conditions. Micrometastatic foci were isolated by microscopic forceps under a stereomicroscope and identified by a GFP+ signal with a fluorescence microscope. Primary tumor tissues were obtained from the orthotopic transplantation region. After discarding nonfluorescent tissues, tumor tissues were cut into small pieces (∼1 mm^3^) by microscissors. The tissue was then suspended in 3 ml DMEM containing 10% (vol/vol) FBS in the presence of 1 mg/ml (wt/vol) collagenase IV (Gibco) with 75 U/ml DNAase I (Sigma‐Aldrich) in a 50‐ml centrifuge tube and digested for 2 hours in a shaking incubator at 37°C at a shaking speed of 400 rpm. The cell suspension was then filtered through a 70‐μm filter mesh to remove connective tissue, and cell clumps were digested with 0.25% trypsin for 5 minutes. Cultured medium was added to stop trypsin digestion, and the cell suspension was centrifuged at 1,200 rpm at 4°C for 5 minutes. The digestion medium was discarded, and the cells were seeded onto 60‐mm culture dishes in 3 ml complete medium at 37°C in a 5% CO_2_ incubator for 3 days. After 10 days of culture, the cells were harvested, and GFP+ cells were isolated by fluorescence‐activated cell sorting for further studies. We used cells at passage 2‐3 for gene expression experiments and MSC coculture tests.

### Xenograft Tumor Model and Administration of MSCs

For HCC tumor mouse models, 5 × 10^6^ RFP‐HCCLM3 cells in 100 μl PBS were injected under the right armpit of nude mice (*n* = 8). We performed the tumor block transplantation method in a sterile environment as previously described 21. Briefly, a tumor mass obtained from the subcutaneous implant region was cut into small cubes sized ∼1 mm^3^. Recipient mice (*n* = 8) were anesthetized with ketamine/xylazine (100 mg/10 mg per kg, Sigma‐Aldrich, St. Louis, MO). The abdomen was disinfected with betadine scrub and 75% medical alcohol. After opening a small subcostal incision, the left lateral lobe of the liver margin was exposed and extracted. The tumor cube was then implanted through a tiny superficial incision of the liver. The incision was closed with a 7‐0 suture to prevent early peritoneal dissemination of tumor cells. For metastatic lung tumor mouse models, RFP‐HCCLM3 cells (2 × 10^6^) in 200 μl PBS were intravenously (i.v.) injected into the tail vein of nude mice and allowed to inoculate the lung (*n* = 8). After 4 weeks of tumor establishment, 1 × 10^6^ GFP‐MSCs were injected into the caudal vein and measured by in vivo flow cytometry.

### In Vivo Flow Cytometry

To monitor the dynamics of GFP‐MSCs in peripheral blood, we performed in vivo flow cytometry as described in a previous study 21. Briefly, an artery about 50–70 μm from the left ear was selected using transillumination with a 535 ± 15 nm light‐emitting diode to observe the vasoganglion. Light from a 488‐nm laser was modulated to a narrow slit across the chosen artery. The beam size at the focal plane was approximately 5 × 72 μm. The depth of focus on the region of interest (i.e., the full width at half maximum of the light slit projected onto the sample in the axial direction) was approximately 50 μm. The passage of fluorescence‐labeled cells through the focused laser slit produced a fluorescence signal that was detected with a photomultiplier tube and sampled at a rate of 5 kHz with a data acquisition card.

### Immunohistochemistry and Immunofluorescence

To assess the recruitment of GFP‐MSCs to the tumor region and other tissues, staining with specific markers was performed. The tissue samples were fixed in precooled isopentane and stored in a −80**°**C refrigerator. Frozen tissues were embedded with Optimal Cutting Temperature and sliced into 8‐μm cryosections, which were then stained with rat anti‐GFP and rabbit anti‐RFP antibodies (Abcam, Cambridge, U.K.) followed by Alexa Fluor 488‐ or 594‐labeled secondary antibodies. After rinsing three times in PBS, the sections were mounted with 4′,6‐diamidino‐2‐phenylindole medium and observed under a fluorescence microscope (Leica, Solms, Germany). To identify micro metastasis, frozen sections were stained with hematoxylin‐eosin (H&E) solution and examined by microscopy.

### RT‐PCR

Total mRNA was obtained and transcribed to cDNA using the RevertAid First Stand cDNA Synthesis Kit K1622 (Thermoscientific, Lithuania) according to the manufacturer's instructions. Quantitative RT‐PCR was conducted with a SYBR Premix Ex Taq Kit (Takara) on an ABI7900HT system (ABI, Foster, CA) with the following conditions: 95**°**C for 30 seconds, followed by 40 cycles of 95**°**C for 5 seconds and 58**°**C for 30 seconds. mRNA expression levels of the target genes were normalized using endogenous GAPDH and calculated based on the 2^−ΔΔCt^ method. Values were expressed as fold change relative to the control group. Experiments were performed four times for each sample.

### Statistical Analysis

Statistical analysis was performed using GraphPad Prism software (GraphPad Software, San Diego). Numerical data are presented as mean ± SD unless otherwise stated. Multiple groups were analyzed by ANOVA followed by Student's *t* tests for pairwise comparisons. Statistical significance was set at *p* < .05.

## Results

### GFP‐MSCs Show Typical Surface Markers and Multipotent Differentiation Capacity

**Figure 1:**
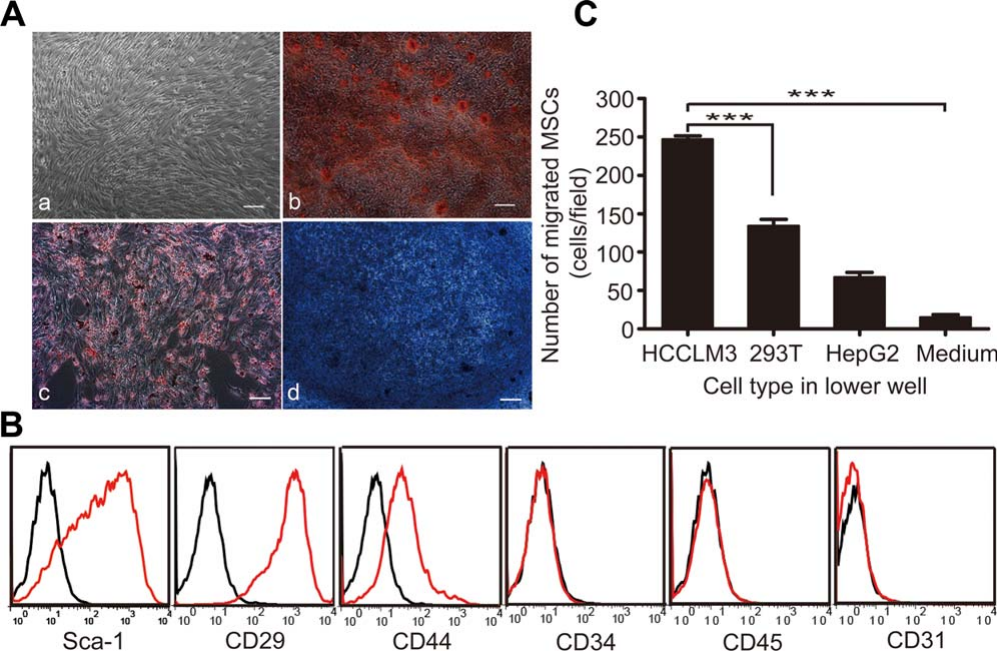
MSCs show typical characteristics and tropism to HCCLM3 cells in vitro. **(Aa)**: Spindle‐shaped morphology of MSCs generated from adult mouse bone marrow. Differentiation capacity of MSCs into **(Ab)** osteoblasts (Alizarin Red S), **(Ac)** adipocytes (Oil Red O), and **(Ad)** chondrocytes (Toluidine Blue). Scale bar: 200 μm. **(B)**: Transwell assay showed a greater migration of MSCs toward GFP‐HCCLM3 cells than toward HepG2 cells (control: 293T cells), ***, *p* < .001. **(C)**: Cell surface markers of mouse MSCs. Histograms showing the expression of surface markers were plotted against controls. Abbreviations: MSCs, mesenchymal stem cells; Sca‐1, stem cell antigen; GFP, green fluorescent protein.

The isolation and purification of bone marrow‐derived MSCs is difficult due to low MSC counts (i.e., 2–5/10^6^ bone marrow nucleated cells) in mouse bone marrow, which contains large amounts of non‐MSCs and hematopoietic cells 27. Therefore, we verified the features of MSCs using standard identification procedures. MSCs isolated from mouse bone marrow exhibited the growth of colonies with spindle‐shape morphology in tissue culture (Fig. 1Aa).

To verify the purity of MSCs, we analyzed cell surface markers by conventional ex vivo flow cytometry. According to the International Society for Cellular Therapy, MSCs express high levels of CD29, CD44, and Sca‐1 and are negative for the endothelial, primitive hematopoietic, and leukocyte antigen markers CD31, CD34, and CD45, respectively, 28. We observed a pattern of MSC surface marker expression that was consistent with this characterization (Fig. 1B).

We further verified the tri‐lineage mesenchymal differentiation capacity of MSCs under in vitro tissue culture‐differentiating conditions. After 14 days of incubation in adipogenic differentiation medium, approximately 90% of cells stained positive for Oil Red O, indicating that GFP‐MSCs exhibited an adipocyte phenotype (Fig. 1Ac). Positive staining for Alizarin Red S demonstrated that GFP‐MSCs were capable of osteogenic differentiation after 21 days of culture in osteogenic differentiation medium (Fig. 1Ab). Furthermore, positive staining for Toluidine Blue showed that GFP‐MSCs also exhibited chondrogenic differentiation capacity (Fig. 1Ad).

### MSCs Preferentially Migrate Toward HCC Cells

To investigate whether human HCCLM3 cells can recruit murine MSCs, we performed in vitro transwell assay to monitor the migration of bone marrow‐derived MSCs toward tumor cells. We found that the number of MSCs migrating toward HCCLM3 cells was significantly higher than those in the control groups (Fig. 1C). Therefore, MSCs showed endogenous tropism to HCC cells, which have a high potential for lung metastasis.

### MSCs Have Different Homing Profiles in Healthy and Tumor Mouse Models

Because in vivo flow cytometry can quantify changes in circulating cells over time in a noninvasive manner, we used this technique to investigate whether systemically administered MSCs show different homing profiles in healthy mice and three types of tumor mouse models with subcutaneous, orthotopically transplanted, or metastasized lung HCCLM3 cells.

**Figure 2:**
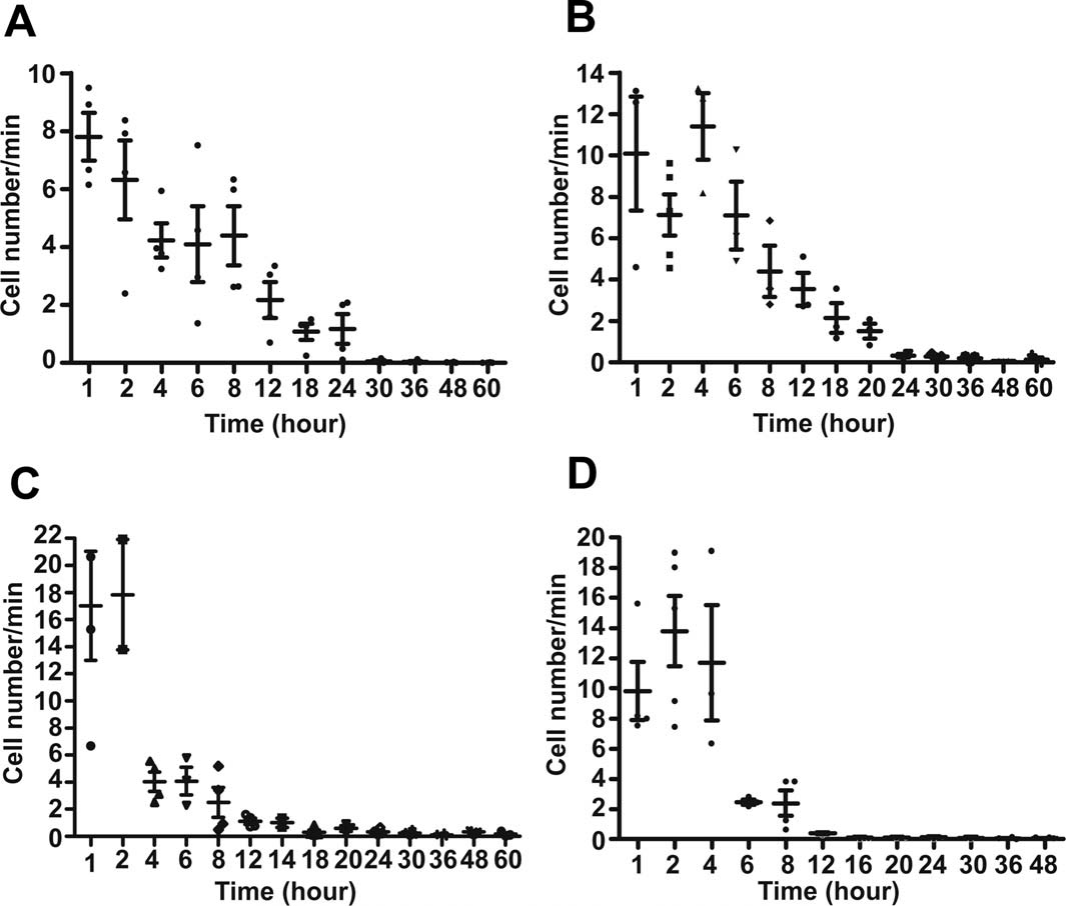
The circulation times of systemically infused mesenchymal stem cells as measured by in vivo flow cytometry were shorter in tumor mouse models than in healthy mice (*n* = 6 per group). The first measurement began within 5 minutes of i.v. infusion and lasted for 2 hours continuously. Later measurements were acquired at the same vessel at 2‐hour intervals until 8 hours and then 6‐hour intervals until 60 hours, with each measurement lasting 1 hour. The number of detected cells per minute is shown as a function of time following infusion in **(A)** healthy mice, **(B)** mice with subcutaneous tumors, **(C)** mice with orthotopic liver tumors, and **(D)** mice with metastatic lung tumors.

The kinetics of systemically infused MSCs in healthy mice may reflect interactions between MSCs and hematopoiesis in the absence of inflammation or tumorigenic cytokines. In healthy mice, the number of GFP‐MSCs steadily declined over a 24‐hour period, becoming scarcely detectable in the bloodstream after 30 hours (Fig. 2A). By contrast, in mice with subcutaneous tumors, the number of GFP‐MSCs initially decreased at 2 hours, increased slightly at 4 hours, and then steadily declined until becoming depleted from the bloodstream after 24 hours (Fig. 2B). After i.v. infusion, most MSCs gradually accumulate in the liver and other organs or become trapped within pulmonary capillaries 17, 18. Therefore, we examined the homing profile of MSCs in mice with HCC cells orthotopically transplanted into the liver. We found that GFP‐MSCs sharply declined in number at 4 hours and became depleted from the bloodstream after 18 hours (Fig. 2C). Finally, in mice with metastasized lung tumors, the number of GFP‐MSCs decreased in the first hour, increased slightly at 2 and 4 hours, and then declined sharply by 6 hours and became depleted after 16 hours (Fig. 2D). Therefore, mice with metastasized lung tumors showed the most rapid depletion of MSCs from the circulation, suggesting that MSCs preferentially home to tissue containing metastasized tumor cells.

Overall, MSCs showed shorter circulation times in tumor mouse models than in healthy mice, suggesting that exocrine factors in the cancer environment promote the localization of MSCs to tumor sites. Furthermore, circulating MSCs may preferentially migrate to tumor sites in the lungs and liver compared with subcutaneous sites.

### Verification of GFP‐MSC Homing Profiles by Conventional Ex Vivo Flow Cytometry

**Figure 3:**
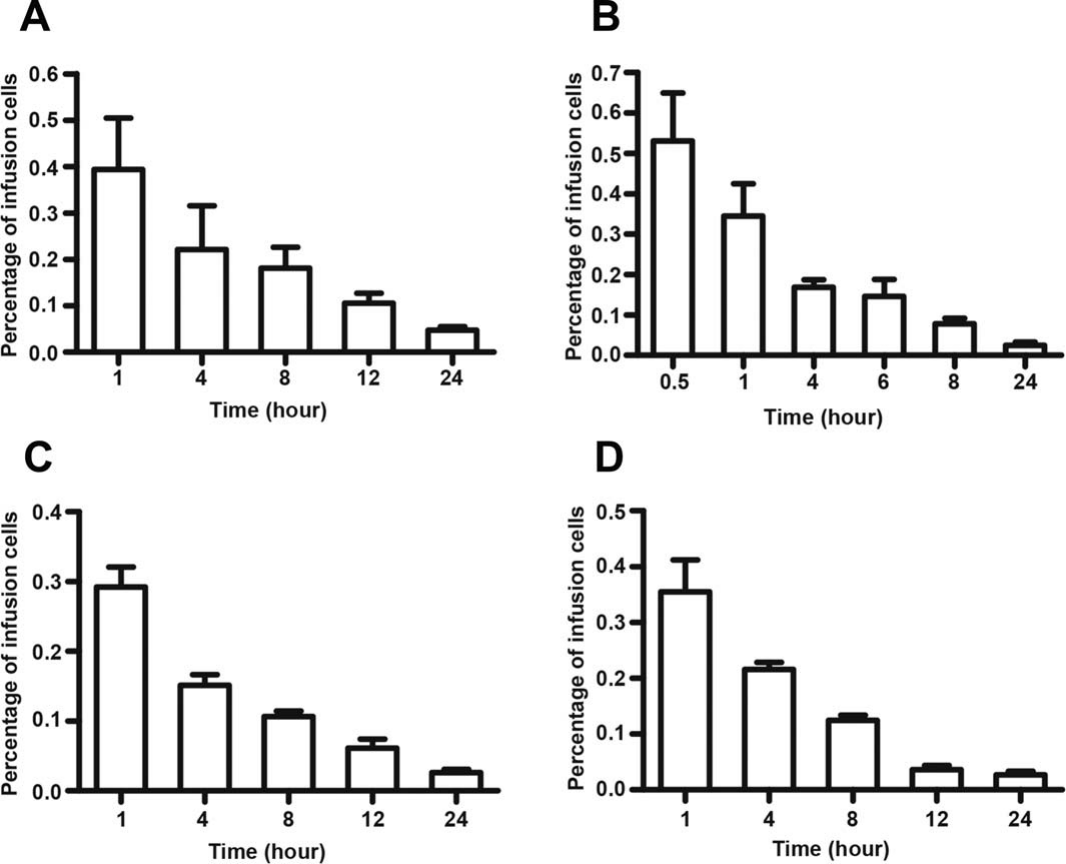
The depletion times of green fluorescent protein ‐mesenchymal stem cells (GFP‐MSCs) in the peripheral blood measured by conventional ex vivo flow cytometry were similar as those measured by in vivo flow cytometry. After MSC infusion into the tail vein, 150 μl blood samples were collected through the orbital venous plexus in **(A)** healthy mice, **(B)** mice with subcutaneous tumors, **(C)** mice with orthotopic liver tumors, and **(D)** mice with metastasized lung tumors at various time points. Data are shown as the proportion of GFP+ cells in 10^5^ sampling cells (*n* = 6 mice per group).

Conventional ex vivo flow cytometry was performed to verify the MSC homing profile measured by in vivo flow cytometry. In mice with subcutaneous tumors (Fig. 3B), orthotopic liver tumors (Fig. 3C), or metastasized lung tumors (Fig. 3D), the proportion of GFP‐MSCs in the peripheral blood steadily declined over 24 hours. In healthy mice, however, the proportion of GFP‐MSCs in the peripheral blood (Fig. 3A) declined at a slower rate than that in tumor‐bearing mice. Therefore, in vivo and conventional ex vivo flow cytometry showed similar MSC homing profiles.

### MSCs Preferentially Migrate to Metastatic Regions

**Figure 4:**
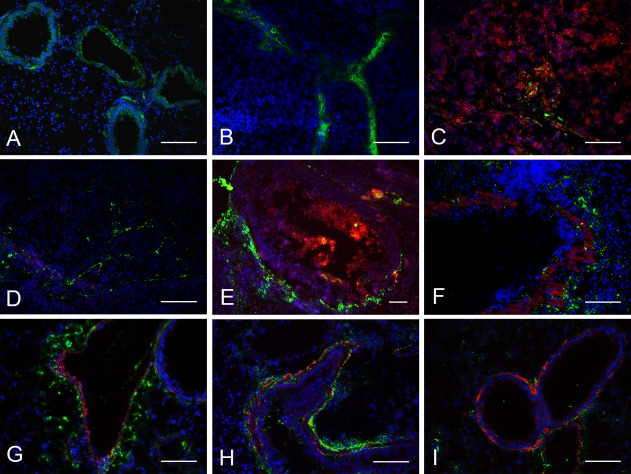
Green fluorescent protein‐mesenchymal stem cells (GFP‐MSCs) migrated to the periphery of red fluorescent protein (RFP)‐HCCLM3 tumors and preferentially accumulated in micrometastatic regions as shown by immunofluorescence staining. In mice with subcutaneous tumors, MSCs became trapped in the **(A)** lungs, **(B)** liver, and **(C)** infiltrated across the subcutaneous tumor site. In mice with orthotopically transplanted tumors, MSCs engrafted to **(D, E)** the periphery of the primary tumor site and **(F)** metastatic regions. **(H**–**I)** In mice with metastasized lung tumors, MSCs preferentially surrounded metastatic regions. Blue: 4′,6‐diamidino‐2‐phenylindole, green: MSCs, red: RFP‐HCCLM3 cells; scale bar: 100 μm.

To examine their targeting sites and biodistribution, MSCs were labeled with the carbocyanine fluorescent dye DiD 29 or EGFP. In mice with subcutaneous tumors, most MSCs migrated to the sinus space in the liver (Supporting Information Fig. S1B) and the lung parenchyma (Supporting Information Fig. S1A) as confirmed by immunofluorescence staining (Fig. 4A, 4B). MSC aggregates were trapped as emboli in the lung and liver during the first 4 hours after infusion through the tail vein (Supporting Information Fig. S2a, S2e), and, over time, smaller numbers of MSCs migrated around the vascular bed (Supporting Information Fig. S2b–S2d) and were randomly scattered across liver lobes (Supporting Information Fig. S2f–S2h). Only a few MSCs migrated to the tumor margin and engrafted to the tumor core during early stages (Fig. 4C and Supporting Information Fig. S2i, S2j). Higher number of GFP‐MSCs engrafted to the subcutaneous tumor region after 12 hours (Supporting Information Fig. S2k, S2l). In mice with orthotopically transplanted liver tumors, MSCs initially engrafted to the periphery of the inoculation region and integrated into the vessel walls, whereas additional MSCs infiltrated into the interior (Fig. 4D–4E) over time (i.e., from 4 to 24 hours; Supporting Information Fig. S3i–S3j). Interestingly, both DiD+ and GFP+ signals widely distributed across intrahepatic metastatic sites (Supporting Information Fig. S1c and Fig. 4F), indicating that MSCs might show stronger tropism toward micrometastatic regions in the liver than toward the primary transplantation site (Supporting Information Fig. S3e–S3g, S3i–S3k). In mice with metastasized lung tumors, RFP‐HCCLM3 cells engrafted around the periphery of the alveolar capillary bed and formed metastatic nodes (Fig. 4H, 4I and Supporting Information Fig. S4a–S4d). The transplanted GFP‐MSCs migrated from the vasoganglion to tumor cell accumulation areas, whereas few GFP+ cells were found in the liver lobes (Supporting Information Fig. S4e–S4h).

The percentage of grafted MSCs in the liver was highest in mice with orthotopically transplanted liver tumors and lowest in mice with metastasized lung tumors (Supporting Information Fig. S1D). Conversely, the percentage of MSCs in lung lobes was highest in mice with metastasized lung tumors and lower in mice with orthotopic liver or subcutaneous tumors. Compared with subcutaneous tumors, mice with orthotopically transplanted tumors had a measurable quantity of MSCs at the primary tumor site.

**Figure 5:**
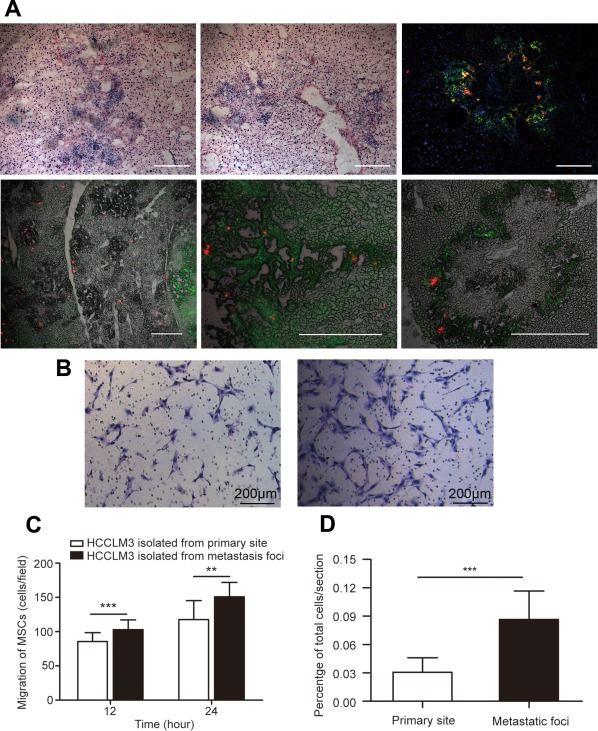
MSCs preferentially infiltrated into micrometastatic regions both in vivo and in vitro. **(A)**: Immunostaining revealed that MSCs homed to liver micrometastatic regions (upper panel). In 5(A), the lower panel shows different magnifications of DiD‐MSCs that migrated to intrahepatic metastatic foci. Scale bar: 100 μm. **(B, C)**: When directly cocultured with HCCLM3 cells in transwell systems, MSCs showed greater migration toward GFP‐HCCLM3 cells extracted from micrometastatic regions (B, the right channel) than toward tumor cells extracted from the primary tumor site (B, the left channel). Scale bar: 200 μm. **(D)**: The number of MSCs engrafted to the primary tumor site and metastatic regions was quantified from 15 random fields per section (three sections per mouse). Data are shown as the proportion of MSCs out of the total number of systemically infused MSCs per section. Original magnification, ×20. **, *p* < .05; ***, *p* < .001. Abbreviation: MSCs, mesenchymal stem cells.

The results of H&E staining combined with immunofluorescence labeling confirmed that MSCs migrated to liver micrometastatic regions (Fig. 5A). Next, we performed in vitro cocultured transwell assay to verify whether MSCs preferentially migrated to GFP‐HCCLM3 cells extracted from the orthotopically transplanted tumor site or those extracted from intrahepatic metastatic areas. In Figure 5B, the left panel shows MSCs that migrated to HCCLM3 cells extracted from the primary tumor site, whereas the right panel shows that a greater amount of MSCs migrated to tumor cells isolated from micro‐metastatic foci. We found that a larger number of MSCs migrated toward cells from liver micrometastatic regions than toward cells extracted from the primary tumor site at both 12 and 24 hours (Fig. 5C). Furthermore, a significantly smaller proportion of MSCs migrated to cells from the primary tumors site than to cells from liver micrometastatic regions (Fig. 5D). These results indicate that MSCs might be more strongly recruited to HCCLM3 cells in metastatic regions than to solid tumors.

### Molecules Secreted by HCC Cells Promote the Homing of MSCs

To investigate the interaction between MSCs and tumor cells from different regions, HCCLM3 cells were isolated from primary tumor sites or micro metastasis regions (Supporting Information Fig. S6), and cultured alone or cocultured with MSCs in a 0.4‐μm transwell chamber. Total mRNA was extracted from the tumor cells to analyze the gene expression of various cytokines, chemokines, and growth factors by RT‐PCR.

**Figure 6:**
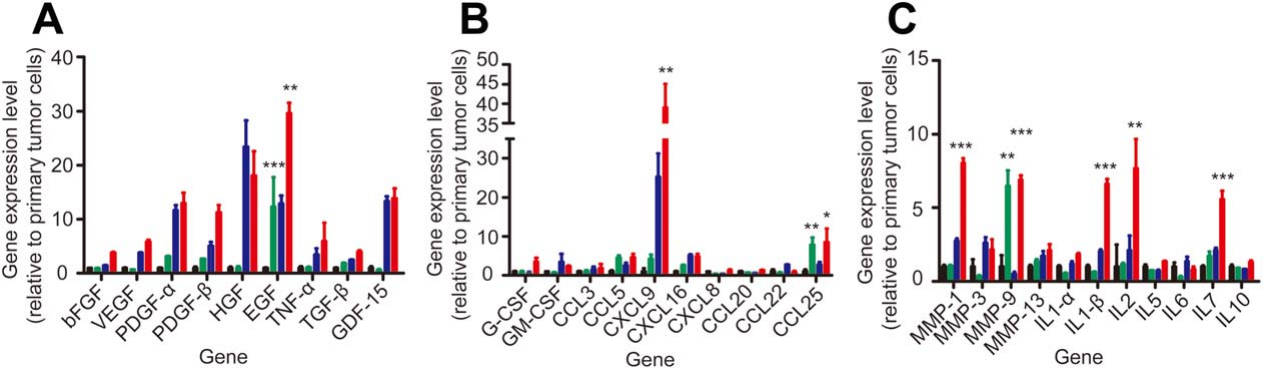
The mRNA expression levels of three cytokines were higher in tumor cells extracted from metastatic regions than in cells extracted from primary tumor sites. mRNA expression of green fluorescent protein (GFP)‐HCCLM3 cells extracted from the primary orthotopic liver tumor site or metastatic regions and cultured alone or cocultured with mesenchymal stem cells (MSCs) in 0.4‐μm transwell systems for 24 hours. **(A)**: Growth factors, **(B)** chemotactic factors, and **(C)** inflammatory factors. Black: HCCLM3 cells extracted from the primary tumor site, green: HCCLM3 cells extracted from micrometastatic regions, blue: HCCLM3 cells extracted from the primary tumor site and cocultured with MSCs, red: HCCLM3 cells extracted from micrometastatic regions and cocultured with MSCs. Data are shown as mean ± SEM; *n* = 3 per group; *, *p* < .05; **, *p* < .005; ***, *p* < .001. Abbreviations: bFGF, basic fibroblast growth factor; CCL, CC chemokine ligand; CXCL, CX chemokine ligand; EGF, epidermal growth factor; G‐CSF, granulocyte colony‐stimulating factor; GDF‐15, growth differentiation factor; GM‐CSF, granulocyte/macrophage colony‐stimulating factor; HGF, hepatocyte growth factor; IL, interleukin; MMP, matrix metalloproteinases; PDGF‐α, platelet‐derived growth factor; PDGF‐β, platelet‐derived growth factor‐β; TNF‐α, tumor necrosis factor; TGF‐β, transforming growth factor; VEGF, vascular endothelial growth factor.

Considering growth factors, HCCLM3 cells from metastatic regions expressed significantly higher levels of epidermal growth factor (EGF) than HCCLM3 cells extracted from the primary tumor site (Fig. 6A and Supporting Information Fig. S5A). This effect was enhanced when HCCLM3 cells were cocultured with MSCs (Fig. 6A). The expression of platelet‐derived growth factor (PDGF)‐α, PDGF‐β, hepatocyte growth factor (HGF), and GDF‐15 increased in both the primary tumor site group and the micro metastasis group when HCCLM3 cells were cocultured with MSCs. These results are consistent with those of previous studies showing that MSCs migrate in response to a variety of growth factors and cytokines including PDGF‐α, PDGF‐β, and HGF 30. Also, coculture with MSCs stimulate HCC cells to express high amounts of GDF‐15, which is a key determinant of MSC tumor tropism 31 and may promote tumorigenesis of HCC cells 32, 33, 34.

Considering chemotactic factors (Fig. 6B and Supporting Information Fig. S5B), the mRNA expression of CCL5, CXCL9, CXCL16, and CCL25 was higher in HCCLM3 cells from micrometastatic regions than in cells from primary tumor sites when cocultured with MSCs. Coculture with MSCs remarkably increased the expression of CXCL9. CXCL9 expressed by HCCLM3 cells might serve as a paracrine factor that promotes MSC recruitment and an autocrine effector that stimulates HCCLM3 invasion and metastasis 35, 36, 37, 38. Whether cultured alone or cocultured with MSCs, HCCLM3 cells from micrometastatic regions expressed higher levels of CCL25 than HCCLM3 cells from primary tumor sites. This result is consistent with previous findings that MSCs exhibit a chemotactic response to CCL25 [39, 40], indicating that the expression of CCL25 from micrometastatic regions might be a leading driver of MSC recruitment to tumor sites. CXCL16, CXCL9, CCL20, and CCL25 have also been found to induce migration of MSCs in a dose‐dependent manner 41. In addition, elevated expression of CXCL16 by prostate cancer cells and breast cancer cells also promotes MSC recruitment 8, 42, 43.

Considering matrix metalloproteinases (MMPs), HCCLM3 cells from metastatic regions showed higher expression of MMP9 than cells from the primary tumor site (Supporting Information Fig. S5C). HCCLM3 cells cocultured with MSCs showed higher expression of MMP1, MMP3, and MMP9 than GFP‐HCCLM3 cells cultured alone (Fig. 6C). These increased levels of MMPs might recruit MSC migration to tumor sites, as MMP1 has been found to mediate MSC tumor tropism through crosstalk with the SDF‐1/CXCR4 axis 44.

Considering inflammatory cytokines, higher levels of interleukin (IL)1‐β, IL2, and IL7 expression were found in HCCLM3 cocultured with MSCs than in HCCLM3 cells cultured alone (Fig. 6C). IL1‐β is an inflammatory cytokine that upregulates the production of MMPs, which stimulates the chemotactic migration of MSCs through the extracellular matrix 45, 46. Elevated expression of inflammatory cytokines stimulated by coculture with MSCs might also enhance the metastasis of HCC cells 47, 48, 49.

In conclusion, GFP‐HCCLM3 cells may recruit MSCs by expressing various cytokines that enhance migration, and MSCs may exert direct paracrine influences on tumor cells, thereby promoting cancer angiogenesis, invasion, and metastasis.

## Discussion

In this study, we investigated the dynamic homing profiles of systemically infused MSCs in healthy mice and three types of tumor mouse models. We use MSCs isolated from murine bone marrow, which is considered a highly heterogeneous population in terms of cell size and surface marker expression. Several studies show that MSCs migrate to sites of injury, ischemia, and tumors by a variety of mechanisms, thus their homing efficiency after systemic infusion could be affected by many different factors. For example, MSCs trapped in mouse lungs after i.v. infusion must overcome major obstacles to exert a therapeutic effect 14, 50, 51. The factors that affect whether MSCs predominately become passively entrapped in small diameter blood vessels or are actively recruited to tumor microenvironments need to be further investigated.

In vivo flow cytometry can be used to quantitatively detect fluorescence‐labeled cells in the circulation in a noninvasive manner. Here, we used this technique to monitor the trafficking and homing profiles of systemically infused MSCs under physiological and pathological states. We found that in mice with subcutaneous tumors, MSCs were completely depleted from the peripheral blood after 24 hours, whereas MSC depletion required 30 hours in healthy mice. In mice with orthotopically transplanted liver tumors, most MSCs were depleted from the bloodstream as early as 4 hours, with complete depletion after 18 hours. However, mice with metastasized lung tumors showed the fastest clearance of MSCs, with complete depletion after 12 hours. Based on these findings, we speculate that the homing of MSCs depends not only on passive entrapment but also on active engraftment to neoplastic tissue. If most MSCs are passively arrested in capillaries or microvessels, circulation times should be similar between physiological and pathological states. Rather, the observed variation in clearance kinetics between tumor mouse models suggests that different paracrine factors released from HCC cells in different tumor sites affect their recruitment abilities. MSC trafficking from the bloodstream to tissues and organs occurs in two stages. In the first stage, MSCs may adhere to or transmigrate from the vasculature depending on the blood supply of the target tissue and the specific cytokines in the peripheral blood. In the second stage, after leaving the circulation, specific cytokines promote MSC engraftment to tumor sites or precancerous lesion areas but not to normal tissue parenchyma. This may explain why MSCs exhibited the longest homing times in mice with subcutaneous tumors, which have poorer blood supply compared with mice with liver or lung tumors. By contrast, mice with metastasized lung tumors exhibited the fastest clearance of MSCs from peripheral blood vessels, which may be due to the combined effect of passive and active homing mechanisms.

MSCs may exhibit an endogenous tendency to engraft to tumors and become part of the tumor microenvironment. In mice with orthotopic liver tumors, MSCs preferentially accumulated at intrahepatic metastatic regions instead of the primary tumor site. Thus, we speculate that MSCs show different patterns of responses to paracrine factors secreted from primary tumor sites and metastatic regions both in vitro and in vivo. By analyzing gene expression, we found that various cytokines were highly expressed when HCC cells were cocultured with MSCs versus cultured alone, which might lead to differences in the homing of MSCs to primary and metastatic regions. In addition, by releasing multiple cytokines into the bloodstream, tumor cells can specifically promote MSC migration 52. On the other hand, MSCs can promote the malignant transformation of tumor cells through soluble factors 8, cell–cell interactions 7, 43, alterations of the extracellular matrix 4, 53, 54, or constitute an early protective niche for the residence of cancer‐propagating cells 55. The observation that MSCs preferentially home to micrometastatic regions suggests that MSCs could potentially serve as a bio‐detector to predict tumorigenesis and tumor development 56.

Based on their inherent tumor‐trophic migratory properties, MSCs are promising anti‐cancer agents that could be used to treat different cancer types 57. After exogenous gene transfection, MSCs can home to tumor sites and express reporter genes that exert targeted anticancer effects. MSCs have been genetically modified to express ILs 58, 59, interferons 60, 61, 62, prodrugs 63, 64, oncolytic viruses 65, 66, 67, and pro‐apoptotic proteins 68, 69. Although some studies provide conflicting data on the trafficking efficiency of systemically infused MSCs to tumor sites, converging evidence indicates that MSCs can produce therapeutic effects by secreting desirable anticancer agents into the bloodstream, either when they become entrapped in nontarget locations or after their engraftment to tumor sites 15. Recently, MSCs have been explored as cell‐based vehicles for delivering therapeutic agents, such as nanoparticles, for targeted tumor therapy 70, 71, 72. However, a number of studies suggest that MSCs are recruited by neoplasia as a source of cancer‐associated fibroblastic cells, creating a favorite niche that promotes carcinogenic progression by secreting paracrine factors, facilitating epithelial‐mesenchymal transition, and increasing metastatic abilities. Because it might be risky to administer exogenous MSCs as drug carriers for long periods of time in tumor environments, it is important to understand MSCs homing profiles and determine appropriate therapeutic time window. We need to know the circulation time of MSCs as vehicles for delivering therapeutic agent to target regions, while avoiding the participation of MSCs in tumor progression. One good approach might be to kill MSCs via suicide gene or therapeutic agents after MSCs home to tumor microenvironment and release drugs. Therefore, an understanding of differences in the homing kinetics of MSCs to tumor sites and the kinetics of drug release from MSCs is important for avoiding systemic side effects. Furthermore, understanding the circulation times of MSCs is necessary for studying the relationship between loading dosages and the pharmacodynamics of MSC‐released agents. For MSC‐encapsulated nanoparticles containing chemotherapeutic or cytotoxic agents, an appropriate drug loading capacity may be critical for preventing the death of MSCs in the circulation and the loss of their tropic properties before homing to tumor sites.

Moreover, whether endogenous bone marrow derived MSCs mobilize into the bloodstream during healthy and diseased state and target to specific tissues needs to be investigated. Our work may provide insights into the homing profiles of endogenous bone marrow derived MSCs both in physiological and pathological condition by using in vivo flow cytometry

## Conclusion

In this study, we characterized the dynamic kinetics of systemically infused MSCs in the peripheral blood in healthy and tumor‐bearing mouse models using in vivo flow cytometry. Our results support the idea that the homing of MSCs to tumor microenvironments involves both passive mechanical trapping and active tumor tropism. After MSCs leave the bloodstream, we found that they tended to engraft to micrometastatic regions rather than primary tumor sites. Moreover, our results suggest that the preferential engraftment of MSCs to metastatic regions is promoted by elevated expression of EGF, CXCL9, CCL25, and MMP‐9 by HCC cells both in vitro and in vivo. These findings increase our understanding of MSC homing mechanisms and interactions with the tumor microenvironment, which can aid in the design of MSC‐based therapeutic strategies.

## Author Contributions

C.‐Y.X. and X.‐B.W.: conception and design; C.‐Y.X., Z.‐R.Y., Y.‐Z.S., D.W., and X.‐B.W.: development of methodology; C.‐Y.X., Z.‐R.Y., and Q.‐Q.C.: collection and/or assembly of data, data analysis and interpretation; C.‐Y.X., Y.‐Z.S., and X.‐B.W.: manuscript writing, review, and/or revision; Z.‐Q.G and X.‐B.W: financial support and final approval of manuscript.

## Disclosure of Potential Conflicts of Interest

The authors indicate no potential conflicts of interest.

## Supporting information

Supporting Information

Supporting Information

Supporting Information

Supporting Information

Supporting Information

Supporting Information

Supporting Information
