# Supplementary figures and images for: Systemically Infused Mesenchymal Stem Cells Show Different Homing Profiles in Healthy and Tumor Mouse Models
Source: Stem Cells Transl Med. 2017 Feb 16;6(4):1120–31. doi: 10.1002/sctm.16-0204 (PMC5442841; doi:10.1002/sctm.16-0204)

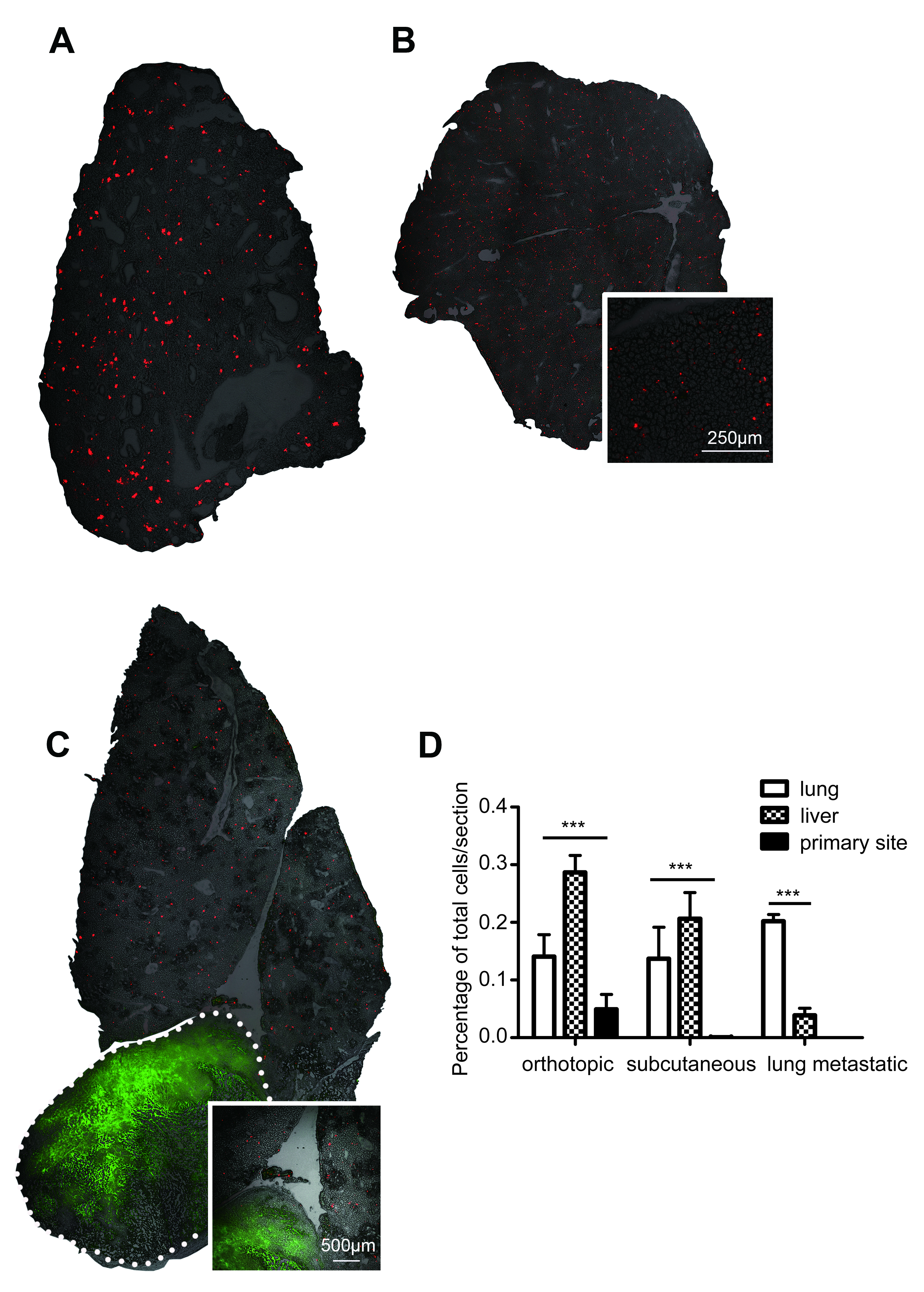

Supplement: Supplementary file 1 — Supporting Information [file SCT3-6-1120-s001.tif]

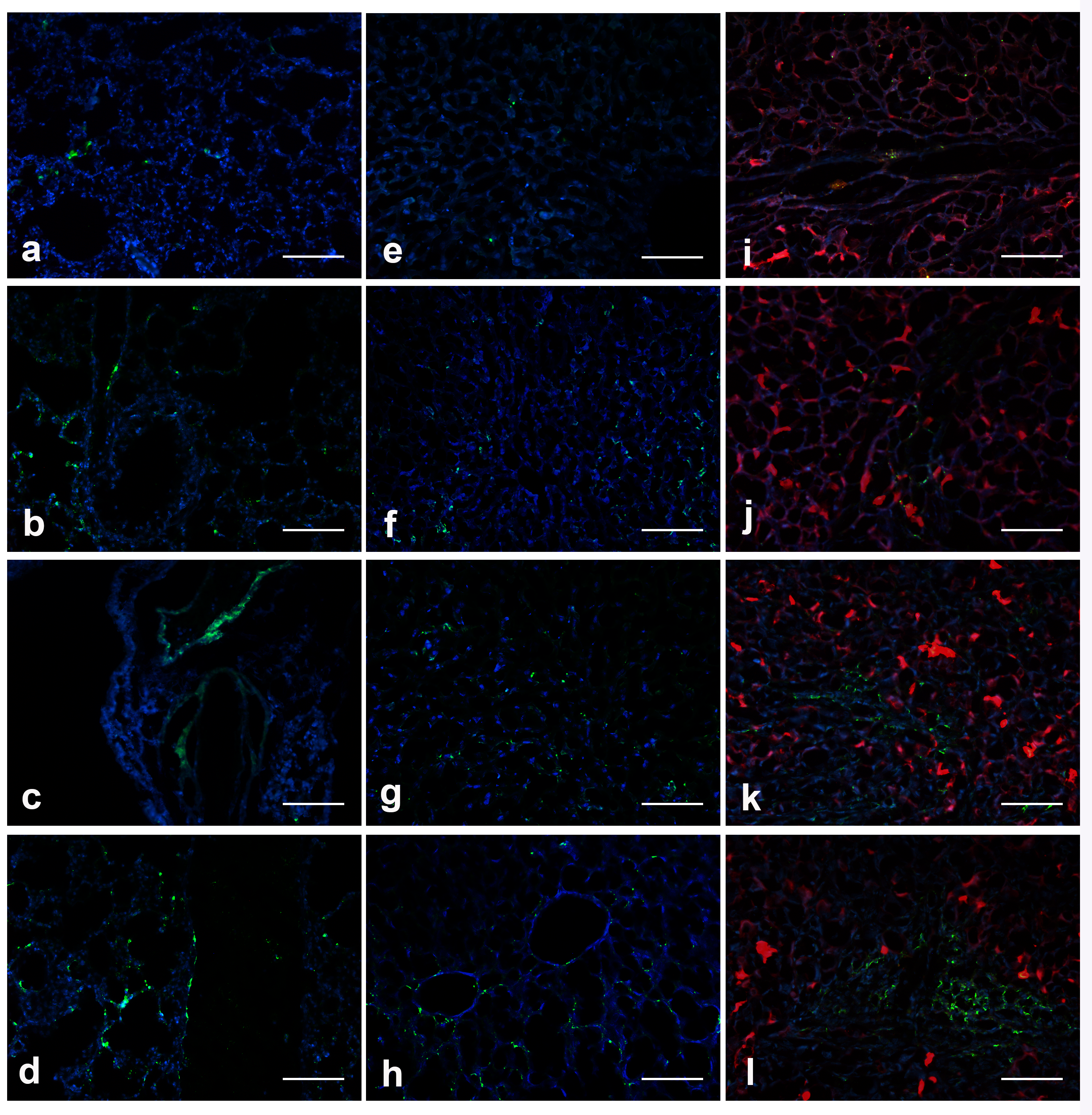

Supplement: Supplementary file 2 — Supporting Information [file SCT3-6-1120-s002.tif]

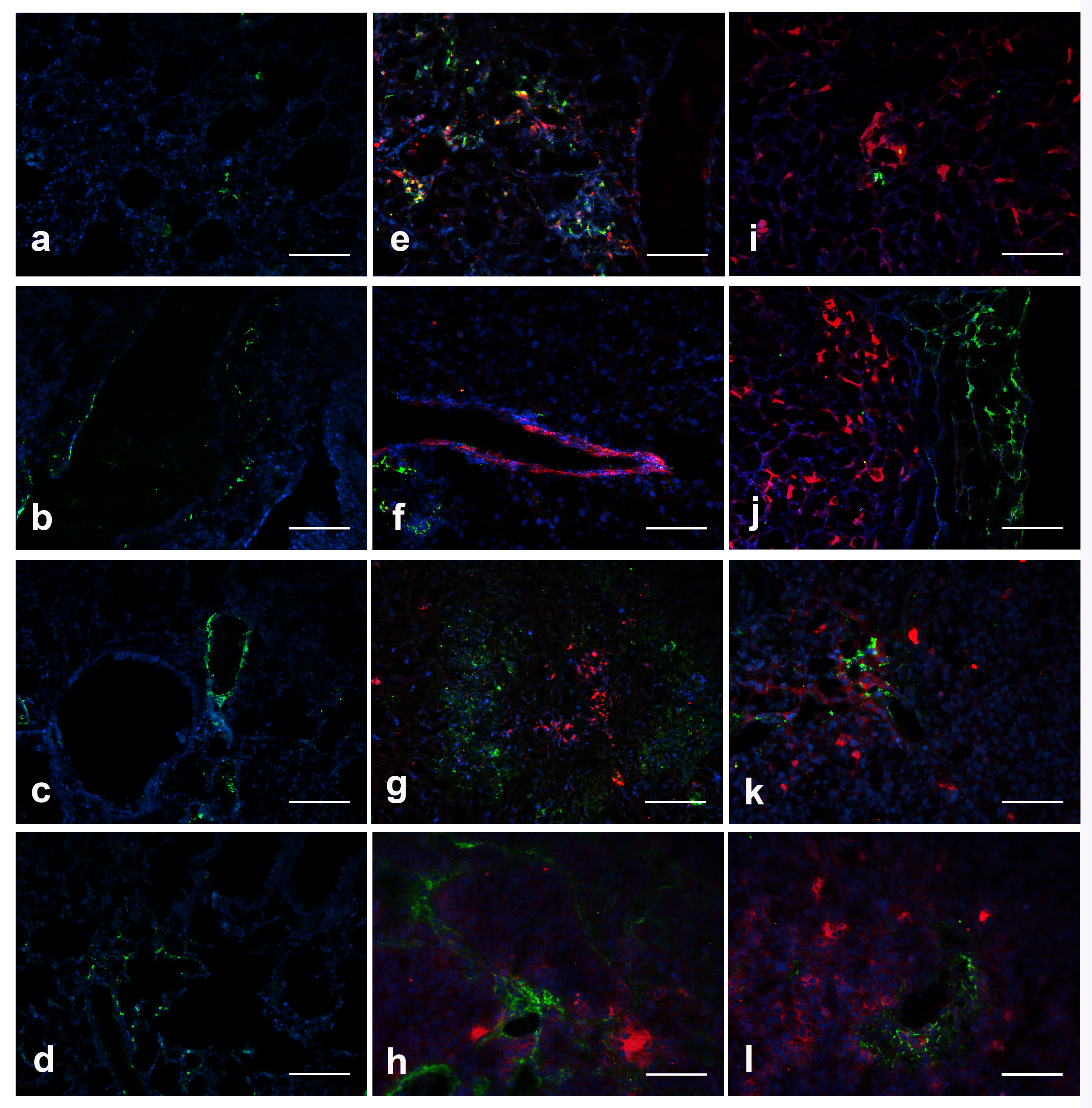

Supplement: Supplementary file 3 — Supporting Information [file SCT3-6-1120-s003.tif]

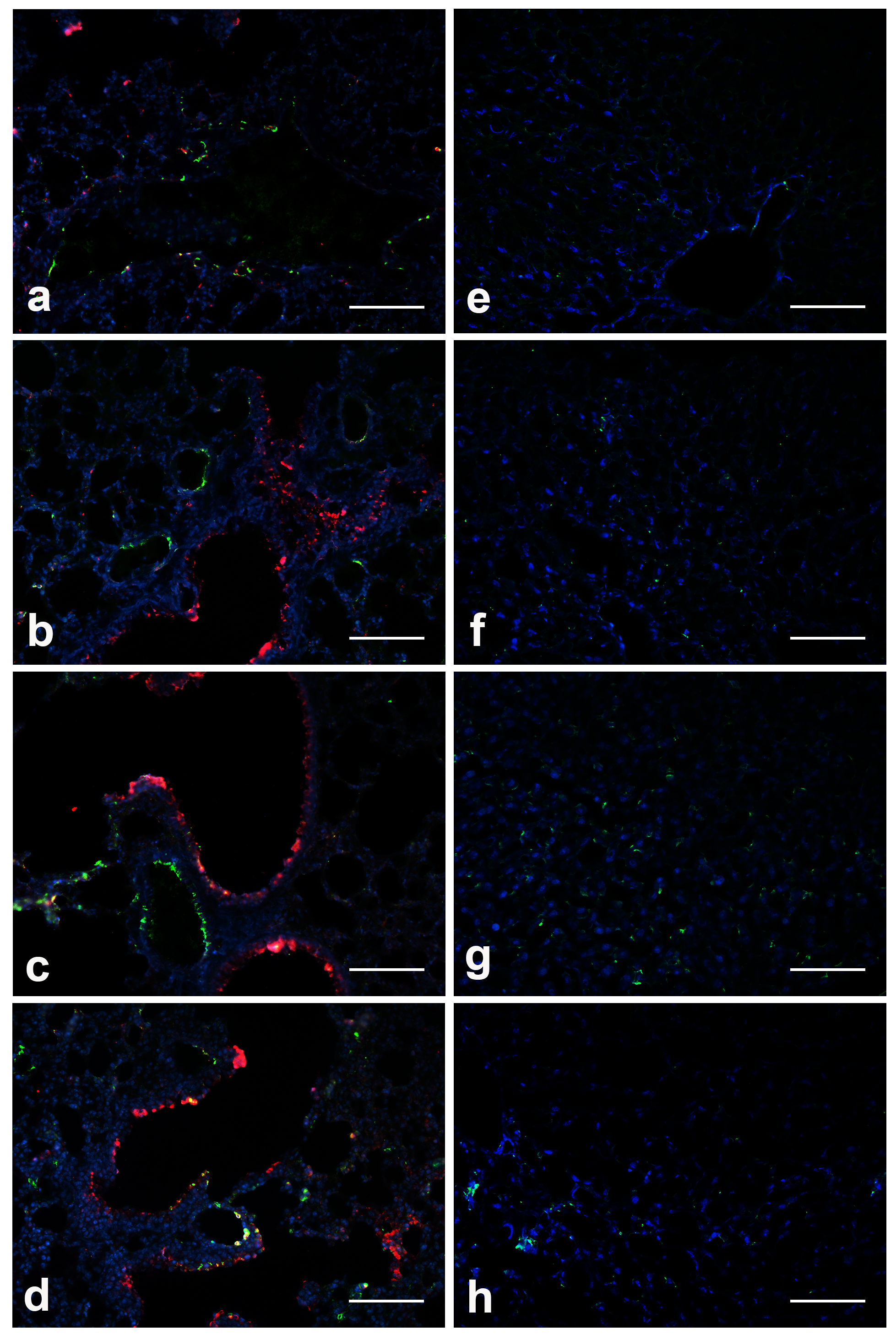

Supplement: Supplementary file 4 — Supporting Information [file SCT3-6-1120-s004.tif]

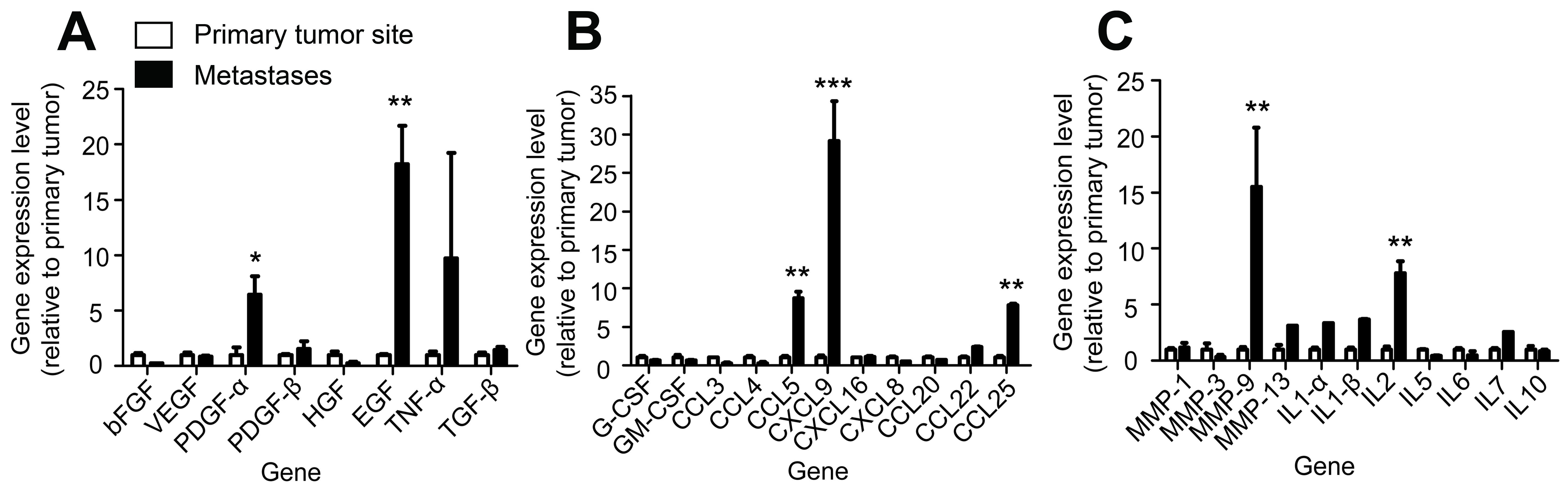

Supplement: Supplementary file 5 — Supporting Information [file SCT3-6-1120-s005.tif]

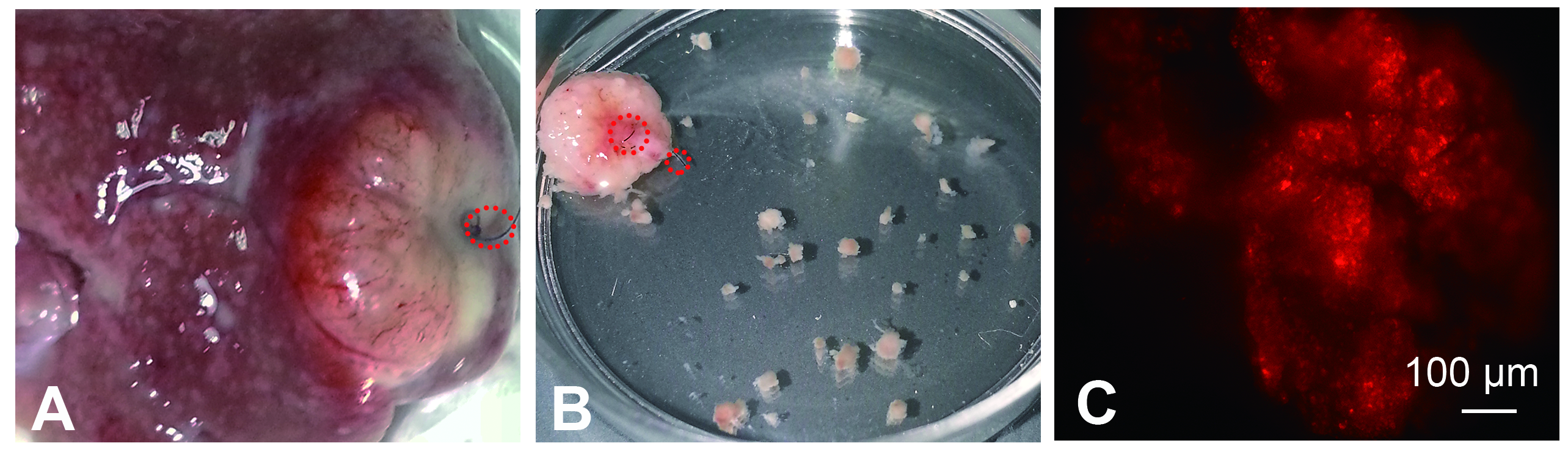

Supplement: Supplementary file 6 — Supporting Information [file SCT3-6-1120-s006.tif]
