# Supplementary material for: Systemically Infused Mesenchymal Stem Cells Show Different Homing Profiles in Healthy and Tumor Mouse Models
Source: Stem Cells Transl Med. 2017 Feb 16;6(4):1120–31. doi: 10.1002/sctm.16-0204 (PMC5442841; doi:10.1002/sctm.16-0204)
Supplement: Supplementary file 7 — Supporting Information [file SCT3-6-1120-s007.docx]

Supplemental Figure 1. The tissue images showed the biodistribution of MSCs in tumor-bearing mouse models. Representative histology of lung or liver lobes showed the distribution of DiD-labeled MSCs after i.v. infusion in mice with subcutaneous or orthotopic liver tumors. (A) A large number of MSCs were trapped in lung parenchyma and surrounded the capillary network. (B) MSCs were scattered in liver parenchyma in mice with subcutaneous tumors. (C) MSCs migrated to the periphery of the orthotopic liver tumor site (in dash line) and specifically engrafted to intrahepatic metastatic regions. Green: GFP-HCCLM3, red: DiD-MSCs. (D) The percentage of MSCs in each tissue section at the same time point (4 days) post-infusion is shown. The number of DiD-labeled MSCs in the engrafted region was quantified from 20 random areas per section (3 sections per mouse). Data are shown as the proportion of cells out of the total number of systemically infused MSCs per section, *** *p* < 0.001.

Supplemental Figure 2. Immunofluorescence staining showed the distribution of GFP-MSCs at different time points in mice with subcutaneous tumors. GFP-MSCs (green) were (a-d) trapped in the lung, (e-h) distributed in liver lobes, and (i-l) infiltrated into the RFP-HCCLM3 (red) subcutaneous tumor site. Tissue sections were obtained (a, e, i) 4 hours, (b, f, j) 8 hours, (c, g, k) 12 hours, or (d, h, l) 24 hours after i.v. infusion. Scale bar: 100 μm.

Supplemental Figure 3. Immunofluorescence staining showed the distribution of GFP-MSCs at different time points in mice with orthotopic liver tumors. GFP-MSCs (green) (a-d) were distributed in the lung, (e-h) accumulated in RFP-HCCLM3 (red) micrometastatic regions, and (i-l) migrated to the primary tumor site. Tissue sections were obtained (a, e, i) 4 hours, (b, f, j) 8 hours, (c, g, k) 12 hours, or (d, h, l) 24 hours after i.v. infusion. Scale bar: 100 μm.

Supplemental Figure 4. Immunofluorescence staining showed the distribution of GFP-MSCs at different time points in mice with metastatic lung tumors. GFP-MSCs (green) were distributed in (a-d) lung metastasis regions (red) and (e-h) liver lobes. Tissue sections were obtained (a, e) 4 hours, (b, f) 8 hours, (c, g) 12 hours, or (d, h) 24 hours after i.v. infusion. Scale bar: 100 μm.

Supplemental Figure 5. The mRNA expression levels are different between primary tumor tissue and metastatic foci. (A) Growth factors, (B) Chemokines, and (C) inflammatory factors. Data are shown as mean ± SEM; n = 3 per group; **p* < 0.05, ***p* < 0.005, ****p* < 0.001.

Supplemental Figure 6. Primary tumor site and micro metastatic regions can be distinguished from the acquired images. (A) Image shows whole liver from a mouse with an orthotopically transplanted tumor. (B) Image shows primary tumor with the end of a suture (in dotted line) and micro-metastatic foci. (C) Metastatic foci are identified by RFP+ signals under a fluorescence microscope.
